# Let the force guide you: a performance-based adaptive algorithm for postural training using haptic feedback

**DOI:** 10.3389/fnhum.2022.968669

**Published:** 2022-11-24

**Authors:** Rakhi Agarwal, Asif Hussain, Varadhan SKM, Domenico Campolo

**Affiliations:** ^1^Department of Applied Mechanics, Indian Institute of Technology Madras, Chennai, India; ^2^School of Mechanical and Aerospace Engineering, Nanyang Technological University, Singapore, Singapore; ^3^Articares Pte. Limited, Singapore, Singapore

**Keywords:** motor learning, task difficulty, postural adaptation, haptic, challenge-point framework, robotic rehabilitation, motor adaptation

## Abstract

Motor learning is an essential component of human behavior. Many different factors can influence the process of motor learning, such as the amount of practice and type of feedback. Changes in task difficulty during training can also considerably impact motor learning. Typical motor learning studies include a sequential variation of task difficulty, i.e., easy to challenging, irrespective of user performance. However, many studies have reported the importance of performance-based task difficulty variation for effective motor learning and skill transfer. A performance-based adaptive algorithm for task difficulty variation based on the challenge-point framework is proposed in this study. The algorithm is described for postural adaptation during simultaneous upper-limb training. Ten healthy participants (28 ± 2.44 years) were recruited to validate the algorithm. Participants adapted to a postural target of 20° in the anterior direction from the initial upright posture while performing a unimanual reaching task using a robotic device. Results suggest a significant decrease in postural error after training. The algorithm successfully adapted the task difficulty based on the performance of the user. The proposed algorithm could be modified for different motor skills and can be further evaluated for different applications in order to maximize the potential benefits of rehabilitation sessions.

## Introduction

Motor skill acquisition can be considered an integral part of human behavior. Therefore, it is crucial to identify an optimal training method to maximize the benefits of motor training sessions. Many factors could influence the process of motor learning. Previous studies have demonstrated the influence of various practice conditions that could have an impact on motor learning. Some of the widely studied factors include extrinsic vs. intrinsic feedback strategies. Many studies have found beneficial effects of extrinsic feedback for implicit motor learning and improving upper limb motor recovery following a stroke (Subramanian et al., 2010). Some studies have also found the learning benefits of self-controlled feedback on a delayed transfer test (Chiviacowsky and Wulf, 2002). Many studies have also evaluated the effect of various socio-cognitive factors on the motor learning. Studies have found that positive social-comparative feedback could enhance the learning of motor skills in a throwing accuracy task (Ávila et al., 2012). Other widely studied factors include error augmentation and errorless training strategies. Many studies have found that error augmentation could promote information processing related to error detection and error correction that are essential for motor learning (Williams et al., 2016). Other studies have found better overall performance after errorless learning as compared to trial-and-error learning (Kessels and Olde Hensken, 2009).

One of the major factors that influence motor learning is task difficulty variation (Christiansen et al., 2018). Typical motor learning and motor rehabilitation paradigms include a sequential progression of task difficulty irrespective of the user’s task performance. However, there is not sufficient evidence in the literature describing a two-way progression of task difficulty, i.e., not just sequentially increasing the task difficulty but also decreasing the task difficulty depending on the user performance. There has been mixed support for such sequential difficulty progression in literature—some studies found evidence that it promotes implicit learning (Maxwell et al., 2001; Capio et al., 2013), whereas other studies did not find similar results (Mount et al., 2007). However, many studies have stressed the importance of adaptive task difficulty variation, i.e., variation of task difficulty based on the performance of the user (Krebs et al., 2003; Choi et al., 2008). Some studies have also reported that adaptivity is essential for the effective transfer of skills (Holmes et al., 2009; Jaeggi et al., 2010). Studies have shown that the learning performance during adaptive schedules outperforms random scheduling (Choi et al., 2008). Matching task difficulty to the learner’s skill level would enhance the learning benefits of the task. Depending on the skill level of the learner, the rate of performance improvement varies from task to task. Therefore, adaptive task difficulty based systems could have widespread application for rehabilitation purposes. Most rehabilitation processes for neuromuscular disorders such as stroke is a time-sensitive process. Studies have shown evidence of “sensitive period” post stroke during which patients show increased responsiveness to training (Zeiler, 2019). Therefore, adaptive scheduling would help to maximize the learning benefits of each rehabilitation session.

In this study, we propose an adaptive algorithm for task difficulty variation based on the performance of the user. The described algorithm is primarily based on the challenge point framework (Guadagnoli and Lee, 2004). According to the challenge point framework, task difficulty can be divided into nominal task difficulty and functional task difficulty. Nominal task difficulty refers to the constant task difficulty irrespective of who performs the task and under what conditions. Functional task difficulty refers to task difficulty relative to the skill level of the performer and environment conditions. Based on the challenge point framework, learning depends on the interpretable information available to the user, which depends on the functional task difficulty of the task. Learning effects could be detrimental if too much or too little information is available. Therefore, there should be an “optimal” level of task difficulty for maximal learning benefit.

We have proposed and verified a task difficulty variation algorithm for postural adaptation during simultaneous upper-limb training based on the above framework. Indirect postural adaptation is studied such that based on the postural error of the trunk, feedback is provided at the reaching task—as opposed to providing direct feedback at the trunk. This kind of postural adaptation is especially useful for reducing trunk compensation during upper-limb rehabilitation in stroke survivors. The basic idea of the algorithm is that task difficulty should be optimal. A task that is too easy to perform would not result in efficient motor learning since mere repetitions do not lead to change in the performance and do not lead to any cortical reorganization associated with motor learning (Plautz et al., 2000). A constant challenge in performing the task is essential for motor learning to happen. On the other hand, when the combined nominal and functional task difficulty is too high, large performance errors could lead to the failure of motor learning (Sanger, 2004). The developed algorithm has been validated for postural adaptation to a predefined postural target using haptic feedback. The current study is an extension of a previous study that shows that haptic feedback could be used for postural adaptation to different postural targets while performing a reaching task (Agarwal et al., 2022). In the previous study, there was no variation in the task difficulty during the training. In the current study, haptic feedback is provided based on the task difficulty computed by the developed algorithm. The proposed algorithm can also be modified for different motor skills in future studies.

## Materials and Methods

### Participants

The developed algorithm has been verified on young and healthy people. Ten healthy participants (seven males and three females) were recruited for this study. The mean (SD) age, height, and weight of the participants was as follows: 28 (2.44) years, 169.21 (5.32) cm, and 71.21 (8.32) kg, respectively. For performing the reaching task, participants used their dominant hand. Hand dominance was assessed using the Edinburgh Handedness Inventory (Oldfield, 1971). All the participants were right-handed. All participants had a normal or corrected-to-normal vision with no history of any neuromuscular injury in the upper extremity.

This study was approved by the Institutional Review Board (IRB) of Nanyang Technological University, Singapore. As per IRB norms, all participants provided written informed consent prior to their participation in the study.

### Experimental setup and protocol

**Figure 1:**
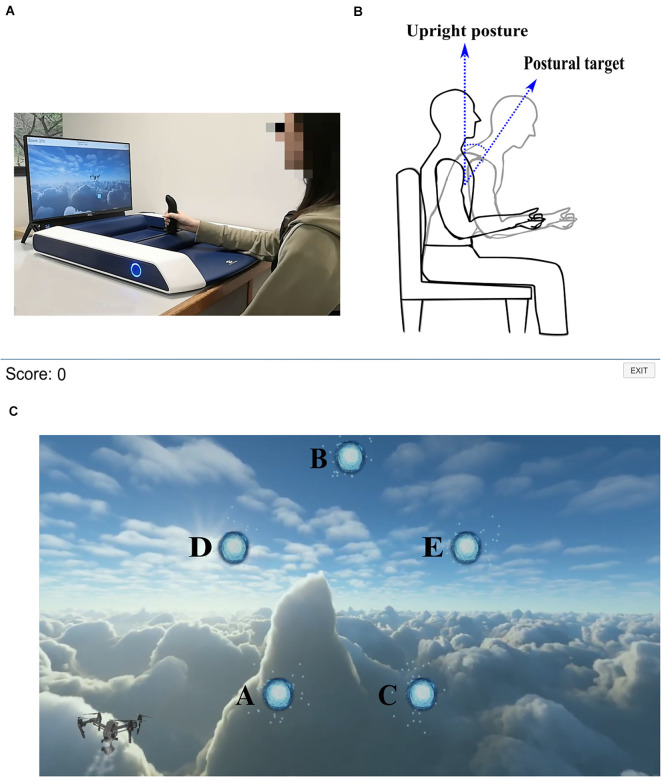
**(A)** H-Man robot: Planar manipulandum called H-Man was used for making reaching movements. **(B)** Postural target: Desired postural target was set as 20° in the anterior direction from the initial upright posture. **(C)** Game screenshot: Point-to-point reaching movement-based game with five visual targets at A, B, C, D, and E.

The experimental setup for this study was similar to the one described in our previous article (Agarwal et al., 2022). Participants were seated in front of a backdrivable, two-degree-of-freedom robotic manipulandum called H-man (manufactured and distributed by Articares Pte. Ltd., Singapore; Campolo et al., 2014). H-Man is an active, clinically validated arm rehabilitation device (Chua et al., 2018). To perform the reaching movement, participants grasped the handle of the H-Man (end-effector of the manipulandum) and moved the handle in a 2-D plane (as shown in Figure 1A). H-Man’s handle movement was replicated as the movement of a cursor on the screen. Additionally, the trunk posture of the participants was measured using Inertial Measurement Units (IMUs). Nine-axis IMUs were used for the study (MPU-9250, Invensense, CA, USA). One IMU was attached to the participants’ chest at the sternum (moving-IMU) using a custom-made strap. The second IMU was fixed at the H-Man (fixed-IMU). Both IMUs were initially calibrated for hard iron distortions by rotating the sensor along each axis. The average of minimum and maximum magnetometer readings was recorded along each axis after rotation to get each axis’s hard-iron distortion correction value. Also, the gyroscope and accelerometer noise were determined from the datasheet of the sensor. A nine-axis Kalman filter was used for sensor fusion and orientation estimation (Zihajehzadeh et al., 2014). The orientation of moving-IMU was measured with respect to the fixed-IMU to get the trunk posture.

The overall objective of the training was the adaptation of trunk posture to 20° in the anterior direction (as shown in Figure 1B). The experimental task for this study consisted of a two-dimensional point-to-point reaching task in a gamified environment. The game was designed using the Unity game engine (Unity Technologies, Denmark). To play the game, participants were required to fly a “drone” to the circles (“visual target”) appearing on the screen. To fly the drone, participants moved the H-Man handle, and the movement of the H-Man handle in the left-right or front-back direction translated to the movement of the drone in the left-right or up-down direction on the screen, respectively. Visual targets appeared at five unique locations on the screen cyclically, in the shape of a star, as shown in Figure 1C. The first visual target appeared at the left bottom of the screen (“Visual target A”). On reaching a visual target, the next one appeared. At any time, only one visual target was visible on the computer screen. Therefore, targets appeared as follows: Visual target A—B—C—D—E—A—and so on. Visual target positions were computed as coordinates of the five-pointed star as follows:


(1) $$\left(x,y\right)=\left(r*\cos\left(2*\pi *k/5+\pi /2\right),r*\sin\left(2*\pi *k/5+\pi /2\right)\right),where\;k=1,...,5$$

Where *r* is the distance of each tip of the star from the center. Therefore, the reaching distance for each trial remained constant throughout the study. Participants’ movement of the drone from the first visual target “A” to the second visual “B” target marked the first trial of the study. Then movement to the next target “C” marked the second trial, and so on.

The study consisted of three phases: pre-training/familiarization phase, training phase, and post-training/test phase. The training phase consisted of 120 trials, while the pre-training and post-training phases consisted of 15 trials each.

### An adaptive algorithm for task difficulty progression during postural training

Trunk postural adaptation to predefined postures while performing a reaching task can be achieved *via* the use of haptic-based feedback (Agarwal et al., 2022). A novel algorithm has been developed to vary the task difficulty during postural training. The developed algorithm is adaptive such that the task difficulty is defined based on the performance of the user. The aim of the algorithm is to “systematically” change task difficulty during motor skill learning based on predefined parameters. The basic idea behind the algorithm is that if the performance of the user during training is consistently “good,” the task difficulty is increased. Conversely, if the user’s performance is consistently “bad,” the task difficulty is decreased. The objective is to keep the user sufficiently challenged during the training session. Therefore, a novel two-way adaptive algorithm has been developed in this study. “Two-way adaptive” implies that depending on the task performance of the user, task difficulty can be increased or decreased during the training.

This study aims to ensure postural adaptation to a particular trunk posture while performing a point-to-point reaching movement. The target trunk posture defines the “nominal task difficulty” based on the challenge-point framework (Guadagnoli and Lee, 2004). If the user deviates from the desired posture, haptic feedback is provided, and the force required to move the handle increases. Therefore, if the users move their trunk towards the desired posture, the haptic feedback decreases, making it easier to move the handle. The deviation of the trunk at any time ‘t’ during the trial is represented as follows: $d_{u}^{t}$ represents the deviation from the upright posture and $d_{d}^{t}$ represents the deviation from the desired posture. However, as part of the adaptive algorithm, task difficulty is varied in terms of a “permissible tolerance” value. Some deviation from the desired posture is allowed, which is termed tolerance. If the trunk posture deviation from the desired posture, $d_{d}^{t}$, is outside the tolerance, i.e., more than the tolerance value, then haptic feedback is enabled. However, if the postural deviation, $d_{d}^{t}$, is within the allowed tolerance, then no extra force is required to move the handle. Therefore, permissible tolerance defines the “functional task difficulty” based on the challenge-point framework (Guadagnoli and Lee, 2004). Permissible tolerance is updated after each trial based on task performance as explained below:

Tolerance is defined as the permissible deviation from the desired posture during the *i*^th^ trial, which is represented by Δ^i^. Trial error for *i*^th^ trial, $e_{tr}^{i}$, is defined as the average deviation from the desired posture during a trial given by:


(2) $$e_{tr}^{i}=\frac{1}{T}\sum_{t=0}^{T}d_{d}^{t}$$

Where *T* represents the total time taken to complete the trial.

Average trial error after *i*^th^ trial is defined as the moving average of last *n* trial errors given by:


(3) $$e_{av_{tr}}^{i}=\frac{1}{n}\sum_{j=i-n+1}^{i}e_{tr}^{j}$$

Where *i* represents the total trials completed, and *n* ≤ *i*.

Task error score is calculated after each trial, *i*. It represents the error outside the tolerance region given by:


(4) $$e_{ta}^{i}=\frac{e_{av_{tr}}^{i}-\Delta^{i}}{\sigma_{h}}$$

Where *σ*_*h*_ represents the standard deviation of the trunk posture for the healthy population.

Based on the task error, tolerance/permissible deviation from the desired posture is updated after each trial as follows:


(5) $$\Delta^{i+1}=\left(1-\gamma \right)*\Delta^{i}+\alpha *e_{ta}^{i}+\beta *\left(e_{ta}^{i}-e_{ta}^{i-1}\right)$$

Where *α* and *β* are the adaptive algorithm parameters that are computed empirically. *γ* represents the slacking factor. Therefore, functional task difficulty is varied during the task based on the performance of the user. So, as part of the algorithm, the tolerance value is updated after each trial based on the performance of the user. Hence, tolerance value is “customized” to the user. If the task performance of the user is improving, task difficulty will increase. However, if the user’s task performance is degrading, the task difficulty will decrease. Hence, this is a two-way adaptive algorithm.

During the rehabilitation of stroke patients, the training objective would be to reduce compensatory trunk movement. Therefore, the desired posture would be an upright posture in this case.

### Haptics based on the developed adaptive algorithm

During the pre- and post-training phases, no haptic forces were enabled. Therefore, participants experienced no extra force while moving the end-effector. During the training phase, haptic feedback was enabled. Therefore, the force required to move the end-effector depends on the participant’s trunk posture. The adaptive algorithm was applied during the training as follows:

### Before the first trial

Task difficulty for each trial was predetermined based on the developed adaptive algorithm. Task difficulty implies the permissible tolerance, Δ. Initial tolerance for the first trial was defined based on the standard deviation of trunk posture for a healthy population as follows:


(6) $$\Delta^{i}=c*\sigma_{h}$$

Where *σ_h_* represents the standard deviation of trunk posture for a healthy population *σ_h_* = 0.92, and *c* is a constant to determine the initial difficulty in terms of standard deviation (c = 12).

The standard deviation of the trunk posture for a healthy population was determined from the baseline measurement of the data collected in the previous study (Agarwal et al., 2022). In that study, 24 healthy participants performed a reaching task using H-Man. During the baseline phase of the study, participants performed reaching tasks freely without the influence of any haptic-based feedback. This baseline phase data have been used to compute the standard deviation of a healthy population to be used in the verification of the adaptive algorithm.

### During each trial

Participants performed reaching movements to the visual target displayed on the screen during each trial while their trunk posture was monitored. If the participants’ trunk posture was detected to be outside the permissible tolerance of that trial Δ*^i^*, i.e., deviation from the desired posture was more than the tolerance value, Δ*^i^*, then haptic feedback was applied. Haptic feedback was applied in the form of a resistive, velocity-dependent force experienced on the end-effector, *F_b_*, in the form of linear damping, *b*, as follows:


(7) $$F_{b}=b*v$$

Where *v* represents the velocity of the movement of the H-Man end-effector.

Damping, *b*, was determined based on a sigmoidal decay function as follows:


(8) $$b=\max\left(b_{m},b_{m}/\left(\left(1+\exp\left(5-\left(d_{d}-\Delta^{i}\right)*10/p_{d}\right)\right)\right)\right)$$

Where *b_m_* represents the maximum damping value (200 Ns/m), *p_d_* represents the desired posture (20 degrees), Δ*^i^* represents the permissible tolerance value of that trial (in degrees), and *d_d_* represents the measured deviation from the desired posture (in degrees).

### After each trial

After each trial, the average deviation from the desired posture during the trial is computed as the trial error using Equation (2). Then average trial error is computed using Equation (3) with a moving average window size of eight trials as follows:


(9) $$e_{av_{tr}}^{i}=\frac{1}{8}\sum_{j=i-7}^{i}e_{tr}^{j}$$

Then, task error is computed using Equation (4), and permissible tolerance is updated for the subsequent trial using Equation (5) with slacking factor, *γ* = 0.2. Adaptive algorithm parameters *α* and *β* are computed experimentally (*α* = 0.45 and *β* = 0.24). The updated tolerance value is used during the subsequent trial, and hence task difficulty is updated after each trial based on the participant’s performance.

### Data analysis

Reaching movement data such as position and velocity of end-effector were recorded from H-Man at 1,000 Hz. Trunk posture orientation data were recorded using IMU at 100 Hz. Postural adaptation performance was evaluated using mean postural error. The mean postural error for each trial was defined as the average deviation from the desired posture, i.e., 20° in the anterior direction. Mean postural error for each trial during pre-training, training, and post-training phases were compared to determine whether haptic feedback-based training resulted in any change in the trunk posture. Adaptive algorithm performance was evaluated based on the change in permissible tolerance value during training. Permissible tolerance was defined as the allowed deviation from the desired posture, beyond which haptic feedback was active. It determined the functional task difficulty of the task. The functional task difficulty (FTD) score, defined as the difference between postural error at the beginning of the trial and permissible tolerance value during that trial, was evaluated to compute the change in task difficulty during the course of training. Change in damping and force at the end effector were also evaluated for the training phase to evaluate the task difficulty in terms of moving the H-Man end-effector. Reaching movement performance was evaluated using the change in velocity of movements during the beginning and end of the training phase. Straightness and smoothness of movements were also compared before and after the postural training (Kamper et al., 2002; Balasubramanian et al., 2015).

Statistical analysis of the data was performed using SPSS (IBM SPSS Statistics, Version 28.0). Friedman ANOVA was performed to investigate the overall difference in mean postural error between pre-training, training, and post-training phases. Wilcoxon signed-rank test was used for pairwise post-hoc analysis to compute the difference in mean postural error between phases. A one-tailed Wilcoxon singed-rank test was used to compute the difference in the following parameters at the beginning and end of the training phase: permissible tolerance, FTD score, force at the end-effector, damping on the end-effector, velocity of reaching movement. The difference in smoothness and straightness of reaching movement before and after the training phase was also evaluated using Wilcoxon signed-rank test. A significance level of 0.05 was chosen for all statistical tests, and Bonferroni correction was applied for multiple comparisons.

## Results

### Mean postural error

**Figure 2:**
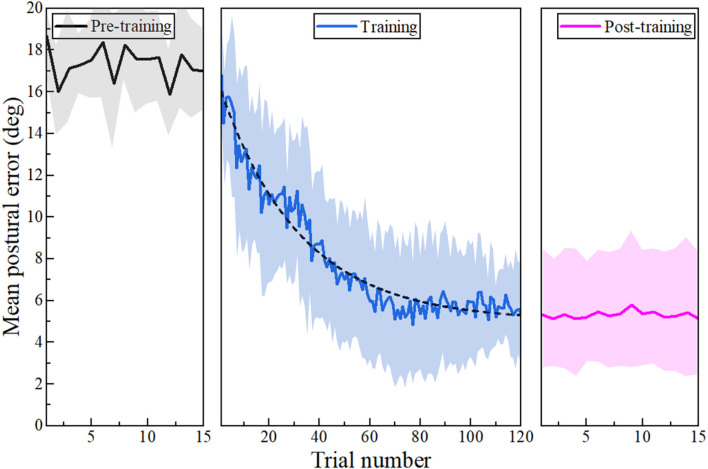
Postural error: Postural error is shown for pre-training (solid black line), training (blue line), and post-training (pink line) phases for each trial. The shaded region around the mean line represents the standard deviation of the postural error. Haptic feedback is enabled during the training phase based on the task difficulty determined by the adaptive algorithm. As a result, postural error decays exponentially during the training phase (dotted black line) compared to the pre-training phase. A decrease in postural error is maintained during the post-training phase after the removal of haptic feedback.

Mean postural error for a particular trial is defined as the average deviation from the desired posture during the trial. The mean postural error during the pre-training, training, and post-training phase of this study is shown in Figure 2. The desired posture in this study is 20° in the forward direction from the initial upright posture. Haptic feedback-based on an adaptive algorithm was enabled during the training phase.

**Figure 3:**
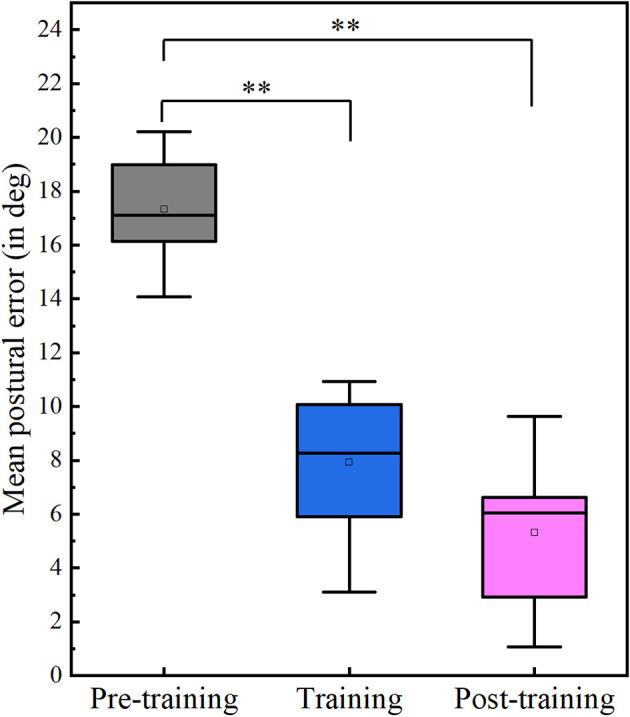
Box plot for change in mean postural error during different phases: Postural error during training and post-training phases is significantly less than in pre-training phases. Double asterisks signify the *p*-value < 0.01. There is no significant difference between the training and the post-training phase (*p* > 0.1).

It was found that the participants’ postural error differed significantly between the three phases of the study (Friedman ANOVA: *p* < 0.001, *W* = 1.00, where effect size estimate W is Kendall’s W value), as shown in Figure 3. Mean postural error decreased during the training phase on the application of haptic feedback. This was supported by post-hoc analysis using the Wilcoxon-signed rank test with Bonferroni correction. It was found that the mean postural error during the training phase was significantly less than the postural error during the pre-training phase (*p* = 0.003, *r* = 0.627, where *r* is the effect size). Moreover, we found that postural error during the post-training phase is significantly less compared to the postural error during the baseline phase (*p* = 0.003, *r* = 0.627), and there was no significant difference between the training and post-training phase (*p* = 0.646, *r* = 0.102). Taken together, this suggests that the participants retained the posture during the post-training phase after the haptic feedback was turned off. Motor learning during training can be modeled using an exponential decay curve of the form:


(10) $$y=A*e^{\left(-\frac{x}{t}\right)}+y_{0}$$

Postural error during training follows this exponential decay curve as shown in Figure 2; where based on Equation (10), *y* denotes postural error during training for each trial, *x* denotes the trial number, and *A, t, y_o_* are computed to be 11.33 ± 0.25, 32.01 ± 1.84, 5.02 ± 0.16, respectively.

### Permissible tolerance value during training

**Figure 4:**
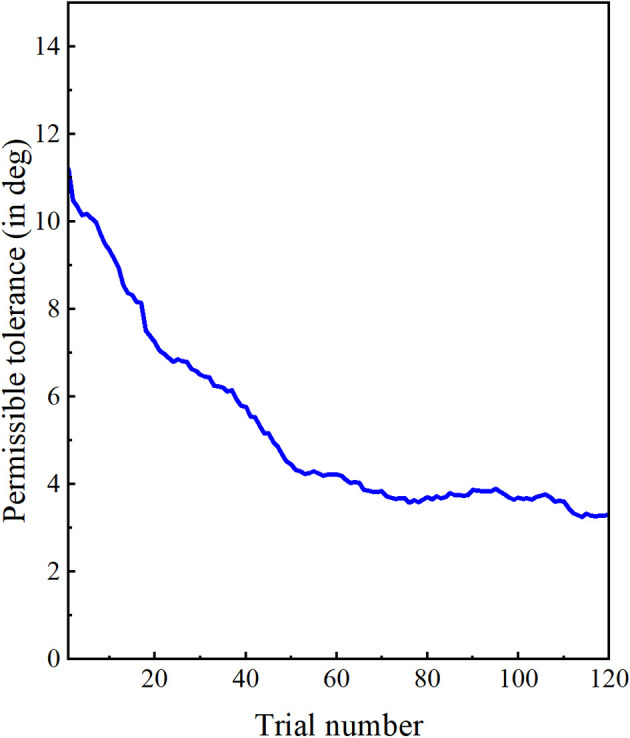
Permissible tolerance: Permissible tolerance value is computed using an adaptive algorithm. The tolerance value decreased during the training phase based on the performance of the participants.

In this study, task difficulty varied during the training phase in terms of permissible tolerance value. Participants received haptic feedback if their postural error was more than the permissible tolerance value computed using an adaptive algorithm. The tolerance value was updated after each trial. The tolerance value during the training phase is shown in Figure 4. We found that the tolerance value at the end of the training phase was significantly less than the tolerance at the beginning of the training phase (*p* = 0.004, *r* = 0.604). Therefore, tolerance value decreased throughout the training as participants adapted to the desired postural target.

**Figure 5:**
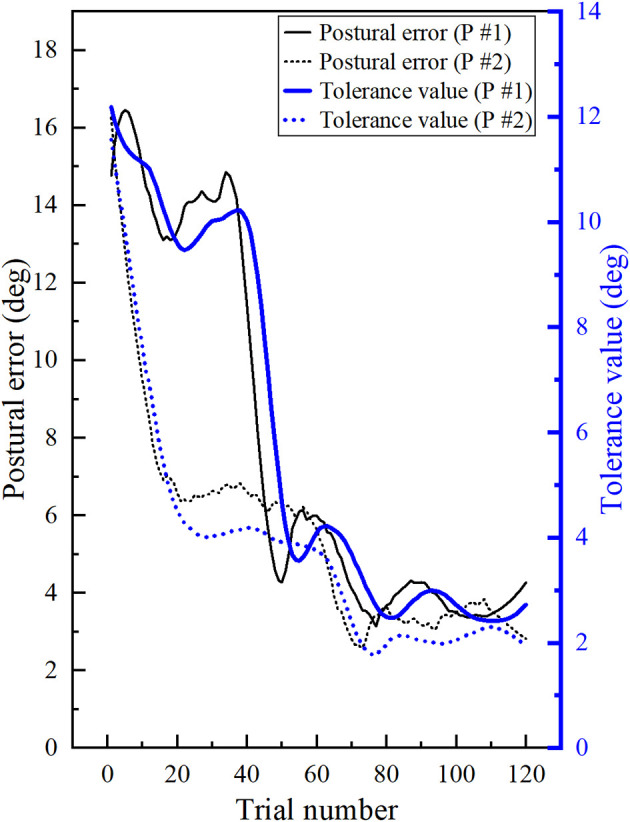
Postural error and tolerance values for two participants: The left (thin, black) axis represents the postural error, and the right (thick, blue) axis represents the tolerance value. Solid lines represent participant number 1, and dotted lines represent participant number 2. Despite similar postural errors at the end of the training, the postural error trajectory is different for both the participants. Computed tolerance values are adapted to the postural performance of individual participants.

We also found a high correlation between the predefined tolerance value of each trial and the postural error during that trial (Pearson correlation, *r* = + 0.9768, *p* < 0.0001). Therefore, the tolerance value was successfully adapted based on the participant’s posture (postural error and tolerance values for two representative participants are shown in Figure 5). We also found that the predefined tolerance value of a particular trial remained significantly less than the participant’s postural error during that trial (*p* < 0.0001, effect size: *r* = −0.614). This implies that the participants always experienced some level of challenge in completing the task during the entire training phase. However, as training progressed, the distance between the tolerance value and the participant’s postural error decreased. The functional task difficulty (FTD) score is defined as the difference between postural error at the beginning of the trial and the predefined tolerance value of that trial. At the end of the training, the FTD score was significantly less than at the beginning (*p* = 0.003, effect size: *r* = 0.615). The functional task difficulty decreased as the training progressed and participants adapted to the desired postural target.

### H-Man force and damping during training

**Figure 6:**
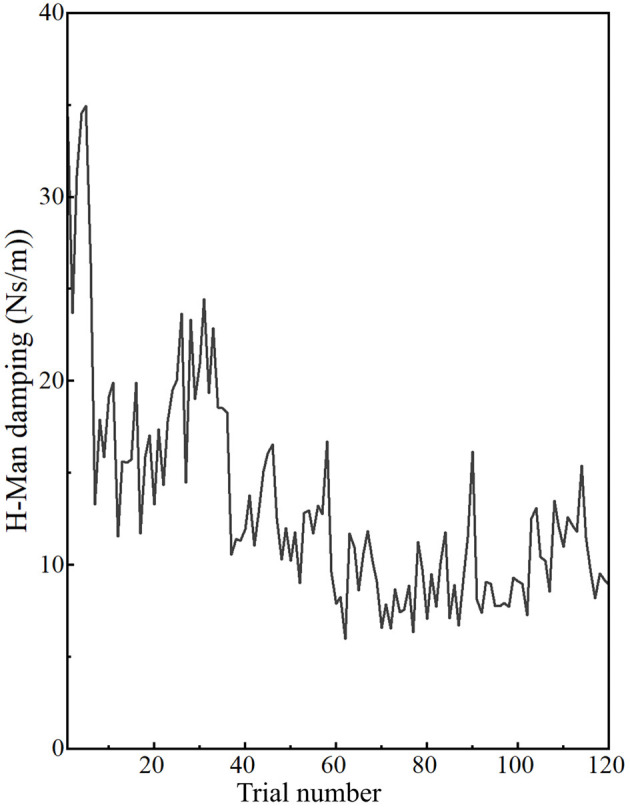
Damping at the end-effector: as participants adapted to the desired postural target, damping at the end-effector decreased during training. Overall damping varied between 5.99 and 34.96 Nm/s.

In this study, participants experienced haptic feedback in the form of damping if their postural error exceeded the tolerance value. Maximum damping was set to 200 Nm/s. Mean H-Man damping during each trial in the training phase is shown in Figure 6. Overall, the damping experienced by the participants’ during training was much lower than the maximum damping force. Participants tried to adjust their posture to minimize the damping force during the training. Mean damping at the end of the training was significantly lower as compared to the damping at the beginning of the training phase (*p* = 0.003, effect size: *r* = 0.615). As the training progressed and participants adapted to the desired posture, the difference between postural error and tolerance value decreased, and the damping force also decreased.

Force experienced by the participants at the H-Man handle also decreased as training progressed. Force due to damping at the end of the training was significantly lower as compared to the force experienced at the beginning of the training (0.026, effect size: *r* = 0.433). Therefore, as the training progressed and participants adapted to the required postural target, performing the task became easier due to the lower damping force.

### Reaching movement performance

The primary task of the study comprised of making reaching movements using the end-effector. We found that the speed of reaching movements was significantly greater at the end of the training as compared to the speed at the beginning of the training (*p* = 0.003, effect size: *r* = −0.615). During the course of training, as participants adapted to the desired postural target, they performed reaching movements at a faster speed. The trial duration reduced significantly at the end of the training phase compared to the beginning (*p* = 0.003, effect size: *r* = 0.615). As participants started adapting to the desired posture, reaching movements became faster, and the time taken to complete each trial decreased.

The smoothness of the reaching movement did not change significantly before and after the training (*p* > 0.5). Moreover, there was no significant change in the straightness of reaching movement before and after the haptic intervention (*p* > 0.5). Therefore, there was no significant improvement or deterioration in the reaching movement performance during postural training.

## Discussion

In this study, we have developed a novel two-way adaptive algorithm for postural adaptation applications. The primary objective of this study was to verify the proposed algorithm for healthy people. Healthy people played a reaching task based game while their posture was continuously monitored. If they deviated from the desired posture, they received haptic feedback-based on the task difficulty determined by the adaptive algorithm.

Previous studies have shown that haptic feedback-based methods can be effective to improve motor learning outcomes (Basalp et al., 2021). Vibrotactile feedback has also been proven effective in virtual motor learning (Islam and Lim, 2022). A previous study has also shown that haptic feedback can be used for indirect postural adaptation applications while performing a one-dimensional reaching task (Agarwal et al., 2022). A similar feedback strategy is used in the present study in combination with task difficulty variation. In the present study, task difficulty was determined using an adaptive algorithm in the form of a permissible tolerance value. The participants received the haptic feedback only if their posture was outside the permissible tolerance values. Here, speculation is that continuously changing the task parameters could lead to an effect of contextual interference. Previous studies have shown that high contextual interference could degrade task performance during skill acquisition (Brady, 1998; Barreiros et al., 2007). Therefore, it is essential to verify that there is no negative interference due to the algorithm and that there is an overall postural adaptation during training. The average deviation from the desired posture is shown in Figure 2. It is evident that there is an overall decrease in postural error during the training. The postural adaptation is maintained even after switching off the haptic feedback. Therefore, there was no negative interference of any kind due to the task difficulty variation. The force feedback related to the participant’s trunk posture guided the participants towards the desired posture.

The objective of using such an adaptive algorithm is to set some level of “optimal task difficulty” (Guadagnoli and Lee, 2004). The nominal task difficulty in this study remained constant, i.e., adaptation to the desired postural target of 20° in the anterior direction while performing the reaching tasks. Higher task difficulty could lead to a feeling of frustration resulting from a lack of success at the desired task. Previous studies have shown that this kind of frustration from high task difficulty could negatively impact the motor learning outcome (Marteniuk, 1976; Sanger, 2004). In this study, functional task difficulty changed based on the permissible tolerance value. The resulting functional task difficulty is designed to always remain lower than or equal to the nominal task difficulty, i.e., permissible tolerance values will always be positive. Moreover, previous studies indicate that the task difficulty should not be lower than the participant’s performance levels for a prolonged period of time, i.e., there should be a sustained challenge for the participants while performing the task to ensure motor learning (Lövdén et al., 2010; Christiansen et al., 2018). Results of the present study suggest that the permissible tolerance value always remained lower than the Participants’ postural error resulting in a continuous challenge during the motor training.

The overall objective of the training is to ensure motor adaptation to the nominal task difficulty level. This study shows that the permissible tolerance decreases during training and slowly starts to reach the nominal task difficulty levels. There is an overall adaptation of the Participants’ posture to the desired postural target. However, the difficulty level is progressively changed throughout the training depending on the task performance of the participant. There is a strong correlation between the predetermined tolerance value for the particular trial and the participant’s postural error during that trial. Therefore, the proposed algorithm ensures that the task difficulty is adapted based on the participant’s performance. To enhance motor learning benefits, the importance of adaptive variation of task difficulty has been emphasized consistently in literature (Zhang, 1994; Choi et al., 2008). An example of the adaptive variation of task difficulty is shown in Figure 5. The postural error and corresponding tolerance values for two representative participants with similar postural errors before and after the training are shown. Despite having a comparable postural error at the end of the training, the trajectory of postural error decay was evidently different between these two participants. As shown in Figure 5, the tolerance value computed using the proposed algorithm is adaptive to the learning curve of the individual participant.

The gradual increment of task difficulty is one of the significant advantages of this algorithm. In our previous study, postural adaptation using haptic feedback was evaluated using sudden perturbation without any variation in task difficulty (Agarwal et al., 2022). In that study, we observed that the postural error quickly reduced to a lower level during the beginning of the training and then remained at that level till the end of the training, leading to a ceiling effect on motor performance. However, overall adaptation was less when compared to the present study. In the present study, the difficulty is changed gradually, leading to an exponential decay of motor error, as shown in Figure 2. There is growing evidence in the literature that gradual perturbation leads to a more complete adaptation and longer after-effects than a sudden perturbation (Kagerer et al., 1997; Michel et al., 2007; Reisman et al., 2007). Studies have shown that the abrupt introduction of high task difficulty leads to large motor errors, and participants are generally aware of the change. However, if rotation is introduced gradually, added error during each increment falls within the bounds of motor noise, and adaptation occurs without awareness (Taylor and Ivry, 2013). This type of “implicit learning” is beneficial for indirect postural adaptation applications due to its lesser dependence on working memory as it frees up attention for other secondary tasks (Poolton and Zachry, 2007). Moreover, individuals who receive gradual training exhibit a slower rate of decay of motor performance, indicating that they adapt more thoroughly as compared to those who receive sudden training (Buch et al., 2003; Huang and Shadmehr, 2009; Sawers and Hahn, 2013). Studies have also shown that not everyone is responsive to sudden training and gradual training leads to better retention as well as a better generalization (Reisman et al., 2010; Musselman et al., 2011). Moreover, the rate of exponential decay of the motor error observed during the training phase in the present study could change when the desired postural target is changed. Future studies could be done to evaluate the change in the decay rate for different target postures.

Speculation is that as participants’ performance at the given task improves during training, they slowly become more “skilled” at the task. There is a fixed upper limit to the task performance in this task, determined by the nominal task difficulty level, i.e., adaptation to the 20° postural target in the anterior direction. In the proposed algorithm, slacking factor plays a part while determining the task difficulty. It works similar to a ramp function while updating the permissible tolerance value for the subsequent trial. This ramp function could theoretically keep decreasing the tolerance value, even if the participant’s performance is constant. Previous studies indicate that there could be a ceiling effect in terms of motor performance, especially for tasks with lower task difficulty (Bonassi et al., 2020; Freidle et al., 2021). To ensure the algorithm’s stability, permissible tolerance should stabilize when the user’s performance approaches ceiling values. In the present study, the functional task difficulty (FTD) score determines the difference between the permissible tolerance value and the participant’s postural error. As Participants’ performance approaches the ceiling values during the course of training, the FTD score slowly decreases towards the end of the training compared to the FTD score at the beginning of the training. Hence, functional task difficulty is not constant throughout the training but instead adapts to a stable value when the participant reaches the upper limit of motor performance during the training task.

The results of this study indicate that towards the end of the training, though participants were able to adapt to the desired posture successfully, the adaptation was not complete. Postural error decreased significantly towards the end of the training compared to the beginning of the training, but the mean postural error at the end of the training was still more than the standard deviation of the healthy population. There could be a multitude of reasons to account for the incomplete adaptation. The force feedback was provided in the form of a damping force. As expected, the damping value and force due to damping decreased as the training progressed. Participants worked towards minimizing the overall force experienced by them at the end-effector of the manipulandum. Near the end of the training, the damping value is only 10.44 N/ms. Speculation is that the low damping force could be below humans’ perceptual capabilities, which would imply a lack of meaningful feedback to improve the motor learning process. Previous studies have reported that human force discrimination capabilities can be degraded for low forces compared to the larger forces (Hatzfeld and Werthschützky, 2012). The smallest change in stimulus intensity to invoke a change in perception of the stimulus is termed as “Just Noticeable Difference” (JND). According to Weber’s law, the ability to perceive a change in the stimulus is directly proportional to the stimulus intensity. However, the perception of the stimulus of low intensity does not follow Weber’s law (Jones, 1989). Furthermore, previous studies indicate that the discrimination threshold of force magnitude is more when the hand is moving than the discrimination threshold for static conditions (Yang et al., 2008). Therefore, high haptic force may be required for feedback to be appropriately perceived in a reaching task and result in motor learning. A lower limit to the damping force value could be established in future studies to ensure a relatively complete adaptation to the desired posture.

The task in this study consisted of playing a reaching movement-based game. Simultaneous postural adaptation could have interfered with the reaching task (Klingberg and Roland, 1997; Herath et al., 2001). Moreover, resistive force feedback-based on postural performance could have further interfered with and diminished the performance of the reaching movement. However, the results of this study indicate no such negative interference of postural adaptation or haptic feedback and reaching movements. In terms of performance at reaching movement, no significant difference was noted for smoothness or straightness of movement before and after the training. Future studies could explore this kind of haptic feedback-based postural adaptation in more challenging tasks such as bimanual reaching movements, 3-dimensional reaching movements, tracing tasks, etc.

Overall, our study indicates that the proposed algorithm could be used to compute functional task difficulty for postural adaptation applications. Haptic force feedback could be used to “gradually” guide the participants towards the desired posture. Our algorithm can easily be modified for learning different motor skills. The postural error can be substituted with other performance metrics for different motor skills. Moreover, in the present version, task difficulty is updated after every trial, which works well for discrete tasks such as reaching movements. The algorithm can also be modified such that task difficulty updates at regular time intervals for continuous tasks such as tracing tasks.

Furthermore, the results of the present study can be evaluated to decrease trunk compensation during the rehabilitation of stroke patients. Stroke survivors often tend to compensate for the impaired upper limb function by moving their trunk (Levin et al., 2002). This is termed “trunk compensation.” Such compensation leads to short-term functional gains but could lead to many long-term issues such as pain, learned non-use, etc. Therefore, therapists aim to reduce such compensatory strategies during rehabilitation, often using trunk restraints (Zhang et al., 2020). But such restraint-based methods are associated with several disadvantages and often require constant supervision of the patients. The present study has evaluated the haptic feedback postural adaptation system for healthy people to a non-upright posture. The experiment task is designed to be purposefully inconvenient. Trunk adaptation to the chosen non-upright posture can be considered an inconvenient posture for the young, healthy participants. Based on the results of the present study, it can be said that participants moved to an inconvenient/unnatural posture rather than applying extra force to counter the provided haptic feedback. This can prove especially useful for stroke rehabilitation since maintaining an upright posture is inconvenient for stroke patients. Moreover, since the target posture is an upright posture during the rehabilitation of the stroke patients, appropriate changes in the current experimental setup would be required to restrict the propensity of leaning on the back of the chair during rehabilitation. Therefore, future studies can explore the efficacy of the algorithm for stroke patients to reduce trunk compensation during rehabilitation.

## Conclusion

We have proposed a novel two-way adaptive algorithm for task difficulty variation for simultaneous postural adaptation during upper limb training tasks based on the challenge-point hypothesis. We have experimentally validated the algorithm for healthy people. Our study shows that the proposed algorithm for task difficulty variation shows promising results. The algorithm controlled the functional task difficulty during training based on the task performance of the participants. Postural error decreased during training, and participants adapted to the desired postural target after haptic feedback-based training. This could be especially suitable for reducing trunk compensation during stroke rehabilitation. However, future studies on stroke patients are required to clearly validate the algorithm for clinical use. Moreover, future studies could modify the proposed algorithm for computing task difficulty during training for different motor skills and validate the algorithm for different applications. Such strategies to maximize the effects of motor learning could substantially enhance the rehabilitation outcomes for several neuromuscular disorders such as stroke, cerebral palsy, etc.

## Data Availability Statement

The raw data supporting the conclusions of this article will be made available by the authors, without undue reservation.

## Ethics Statement

The studies involving human participants were reviewed and approved by The NTU Institutional Review Board (NTU-IRB), Nanyang Technological University, Singapore. The patients/participants provided their written informed consent to participate in this study.

## Author Contributions

RA devised the study design (with inputs from VSKM and DC), performed data collection and analysis, and drafted the manuscript. AH contributed to the study design, interpretation of the results, and coordinated participant recruitment. VSKM and DC established the hypothesis and direction/scope of the study and provided vital feedback about study design, task selection, and interpretation of the results. All authors contributed to the article and approved the submitted version.

## Funding

This research is partly supported by the National Research Foundation, Singapore under its Medium Sized Center for Advanced Robotics Technology Innovation (CARTIN).
